# A Comparison of Clinical and Radiographic Signs of Nontuberculous Mycobacterial Pulmonary Disease, Destructive Drug-Resistant Pulmonary Tuberculosis and a Combination of Nontuberculous Mycobacterium Pulmonary Disease and Pulmonary Tuberculosis

**DOI:** 10.3390/pathogens12070887

**Published:** 2023-06-29

**Authors:** Dmitrii Giller, Galina Scherbakova, Inga Enilenis, Ivan Martel, Oleg Kesaev, Vadim Koroev, Anna Popova, Alexandr Ilyukhin, Valeria Basangova, Sergey Smerdin, Shokirjon Mayusupov, Sergey Saenko, Olga Frolova, Veronika Vinarskaya, Lyudmila Severova

**Affiliations:** 1 Department of Phthisiopulmonology and Thoracic Surgery Named after M.I. Perelman, I.M. Sechenov First Moscow State Medical University (Sechenov University), Moscow 119991, Russia; giller_d_b@staff.sechenov.ru (D.G.); shcherbakova_g_v@staff.sechenov.ru (G.S.); enilenis_i_i@staff.sechenov.ru (I.E.); martel_i_i@staff.sechenov.ru (I.M.); kesaev_o_sh@staff.sechenov.ru (O.K.); koroev_v_v@staff.sechenov.ru (V.K.); popova_anna97@bk.ru (A.P.); ilyukhin_a_n@staff.sechenov.ru (A.I.); lera.basangova@yandex.ru (V.B.); opfrolova@yandex.ru (O.F.); vinarskaya_v_i@student.sechenov.ru (V.V.); 2 State Budgetary Healthcare Institution of the Moscow Region “Moscow Regional Clinical Anti-Tuberculosis Dispensary”, Sukmanikha Village, Shchelkovsky District, Moscow 170555, Russia; 3 Republican Specialized Scientific-Practical Medical Center for Phthisiology and Pulmonology, St. Majlisy, Shaykhotokhur District, Tashkent 100071, Uzbekistan; shokir.may@mail.ru; 4 Rostov Regional Clinical Center of Phthisiopulmonology, St. Orskaya, 24, Rostov-on-Don 344065, Russia; saenkosergey@yandex.ru; 5 Department of Phthisiopulmonology, Pirogov Russian National Research Medical University, Moscow 117997, Russia

**Keywords:** nontuberculous mycobacterial pulmonary disease, pulmonary tuberculosis, NTMPD diagnostic imaging, clinical signs of NTMPD

## Abstract

A misdiagnosis of isolated pulmonary tuberculosis (pTB) is highly likely when a patient has nontuberculous mycobacterial pulmonary disease (NTMPD) or a combination of nontuberculous mycobacterium pulmonary disease and pulmonary tuberculosis. Frequently, bacterial excretion is absent or only Mycobacteria tuberculosis (MBT) is found. This often results in an incorrect diagnosis and subsequent misinformed treatment regimes. In order to determine possible clinical and radiographic differences between patients with NTMPD (Group 1), destructive drug-resistant pulmonary tuberculosis (Group 3) and a combination of NTMPD and pTB (Group 2) we compare clinical and radiographic signs for these three patient groups. When comparing with Group 3 (2.5%), Groups 1 (25%) and 2 (17.4%) have a substantially higher incidence of pulmonary haemorrhages. Thus, upon clinically observing the combination of pTB and NTMPD, there are no pathognomonic clinical and radiographic detected symptoms. However, the presence of an indolent course, hemoptysis and bronchiectasis in the presence of acid-fast bacteria (or identified MBT) in the sputum makes it possible to suspect not simple pTB, but a combination of pTB and NTMPD. To clarify this necessitated in-depth bacteriological examination.

## 1. Introduction

Nontuberculous mycobacteria (NTM) are ubiquitous in the environment and found in most water sources and soils. Furthermore, NTM are conditionally pathogenic. Consequently, a single isolation of these bacteria from the respiratory tract only indicates body media colonisation and does not unequivocally define the presence of disease [1,2,3,4,5,6,7].

The likelihood of misdiagnosing pulmonary tuberculosis (pTB) exists when a patient has a combination of nontuberculous mycobacterial pulmonary disease (NTMPD) and pTB, or simply NTMPD. Doctors usually do not consider that an absence of bacterial excretion or only Mycobacteria tuberculosis complex (MBT) could be presented when a patient has a combination of NTMPD and pTB. In addition, similar clinical and radiographic signs make a differential diagnosis between these two diseases difficult [4,7,8,9,10].

It seems reasonable to evaluate the similarities and differences in the clinical and radiographic manifestations of pTB and NTMPD in combination when comparing isolated extensively drug-resistant fibro-cavitary TB and isolated NTMPD [4,7,8,9,11,12,13,14,15,16,17,18,19,20,21,22,23,24,25]. This may allow the physician to identify situations where additional examinations are required to detect not only MBT but also NTM, since the finding of NTM in sputum, bronchoalveolar lavage, biopsies and operative material forms the basis for a reliable verification of NTMPD.

## 2. Materials and Methods

### 2.1. Study Design

**Figure 1:**
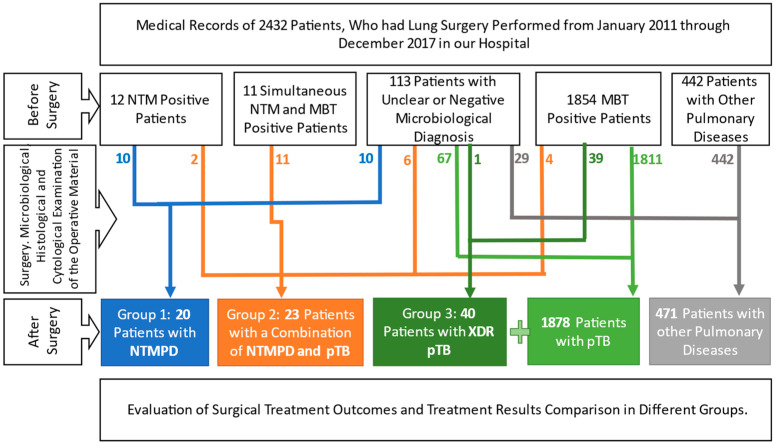
Study Design.

The medical records of 2432 patients who have had lung surgery performed between January 2011 to December 2017 at Sechenov University Phthisiopulmonology Clinical Hospital were analysed. A total of 2432 patient records were examined. Overall, 1918 patients had tuberculosis, 20 NTMPD, 23 with a combination of pTB and NTMPD, and 471 other pulmonary diseases. We included all 20 patients with NTMPD in Group 1, and constructed Group 2 with 23 patients having a combination of NTMPD and pTB. However, during the period under consideration, 311 patients with destructive extensive drug-resistant pulmonary tuberculosis (XDR pTB) were identified, of which 40 patients were selected randomly via a random number selector which operated on a list with numbers corresponding to those assigned for each patient. These 40 patients constituted Group 3 (Figure 1).

We have already presented the main aspects of study design, conservative treatment and surgery in the article “Surgical Treatment of Nontuberculous Mycobacterial Pulmonary Disease and a Combination of Nontuberculous Mycobacterium Pulmonary Disease and Pulmonary Tuberculosis” [26]. In order to compare clinical and radiographic signs of NTMPD, XDR pTB, or a combination of NTMPD and pTB, additional data were analysed. All patients’ radiographic data from disease identification up to surgery were analysed and summarised. At a minimum, one high-resolution CT scan has been performed for each patient prior to surgery. The formation of comparison Group 3 was because patients with XDR pTB are generally considered to be a severe group of patients, who respond with difficulty to chemotherapeutic treatment. Surgery on such patients is associated with greater risk and difficulties than surgical treatment for other forms of tuberculosis [27,28,29,30,31].

Patients in the list for potential inclusion into Group 3 did not include those admitted before January 2011 or after December 2017, those who received lung resections, and those who did not have NTMPD, destructive extensive drug-resistant pulmonary tuberculosis or a combination of NTMPD and pTB. Such patients did not meet our selection criteria.

### 2.2. Treatment Regime

All patients with NTMPD were diagnosed in accordance with the ATS/IDSA recommendations, 2007 [2] and were HIV-negative. Antibiotic treatment was prescribed at the hospital where a diagnosis was established. In some cases diagnosis has been clarified only after surgery. Treatment for Groups 1 and 2 has also been described in “Surgical Treatment of Nontuberculous Mycobacterial Pulmonary Disease and a Combination of Nontuberculous Mycobacterium Pulmonary Disease and Pulmonary Tuberculosis” [26].

Destructive extensive drug-resistant pulmonary tuberculosis was diagnosed according to extensive investigation and the analysis of microbiological, cultural and molecular genetic research methods, radiological data and medical history data. Chemotherapy was prescribed according to Russian Federation government regulations, clinical guidelines and data on individual drug susceptibility of mycobacteria in patients (Table S1) [26,32,33].

A multi-disciplinary medical commission consisting of a pulmonologist, phthisiatrician, thoracic surgeon, epidemiologist, and anesthesiologist arbitrated on the need for lung surgery. Indications for surgical treatment were the ineffectiveness of adequate antibiotic therapy, continued high risk of bacterial excretion or recurrence of bacterial excretion, the presence of cavities in the lung tissue, and complications such as hemoptysis, pneumothorax, and unclear diagnosis.

Postoperative management included the continuation of antibiotic therapy, its correction when new data on drug resistance appeared after an analysis of the operative material, the prescription of seasonal courses of chemoprophylaxis (Russian government regulation [32,33]), and, if necessary, regular observation and annual post-operative X-ray examinations for at least 3 years. Patients diagnosed with pulmonary tuberculosis were registered with a phthisiatrician at their local hospital and received regular follow-up and annual radiographic examinations for at least three years after surgery.

### 2.3. Statistical Analysis Methodology

Statistical analyses were conducted using IBM SPSS Statistics 22.0. The significance of the difference in frequencies was determined via the chi-square test, in a pairwise comparison using the exact version of the test. Due to the small number of patients, when comparing the distributions of scale-type variables in groups, the nonparametric Mann–Whitney test was applied, then the data were presented as medians and quartiles. Confidence bounds for frequency were calculated using the binomial distribution. Differences were considered statistically significant for *p* < 0.05; in multiple comparisons, the possibility of false positive differences being taken into account.

## 3. Results

There are relatively more female patients in Group 1 and Group 2 in comparison with Group 3. Nevertheless, no statistically significant differences when considering age and gender between groups were determined (Table 1 and Table 2). 

**Figure 2:**
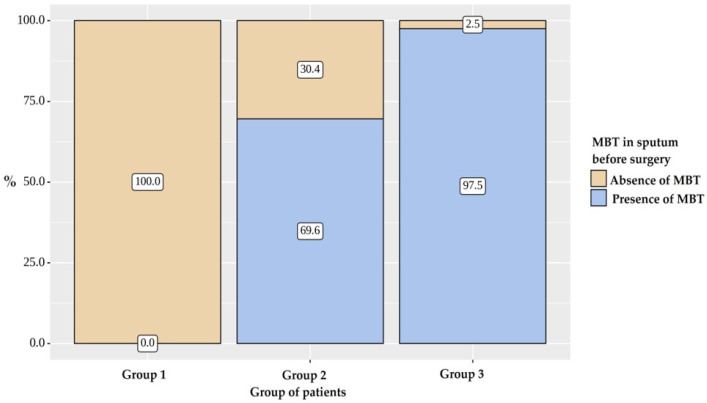
MBT in sputum before surgery.

**Figure 3:**
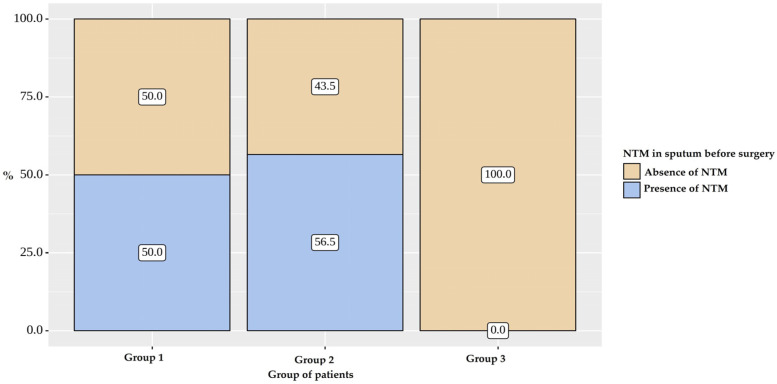
NTM in sputum before surgery.

Bacterial excretion was indicated for 69 (83.13%) patients in all groups. Patients suffer bacterial excretion in Groups 1 and 2 less frequently than in Group 3 (*p* < 0.001) (Figure 2 and Figure 3).

In Group 1, 9 (45%) patients were positive for *M. avium*, 3 (15%) for *M. fortuitum*, 3 (15%)—*M. abcessus*, 2 (10%)—*M. xenopi* and 1 (5%) for *M. kansasii*, *M. chelonae* and *M. simiae*.

In Group 2, 12 (52.17%) patients were positive for *M. avium*, 2 (8.69%) for *M. fortuitum*, 4 (17.39%)—*M. kansasii*, 2 (8.69%)—*M. abcessus* and 1 (4.34%) for *M. chelonae*, *M. simiae*, and *M. xenopi*.

Disease duration prior to surgery in Group 3 was statistically significantly greater than Groups 1 and 2 (Table 3).

Lung cavitary lesions were found in 77 (92.8%) patients. Only single cavitary lesions were observed in 32 (38.6%) patients and multiply in 45 (54.2%). There were 59 (71.1%) patients with unilateral cavitary lesions and 18 (21.7%) with bilateral. The frequency of multiple and bilateral cavitary lesions was less in Group 1 in comparison with Groups 2 and 3. The cavitary lesion diameters primarily ranged from 2 to 4 cm in all three groups of patients (Table 4 and Table 5). More than 10 lung segments were damaged in 19 (47.5%) Group 3 patients, in 5 (25.0%) Group 1 patients and in 5 (21.74%) Group 2 patients.

There were a range of radiographic signs (Table 5, Table 6 and Table 7). Most patients with NTMPD (Group 1) and with a combination of NTMPD and pTB (Group 2) have suffered unilateral lung damage—65.0% and 73.9%, respectively. Bilateral lung damage was observed in most patients with XDR pTB (Group 3, 65.2%). The pathological process was limited (up to three segments) in half of patients in Group 1, in Group 2 the majority (82.6%) suffered a widespread process while in Group 3 the incidence of widespread lung damage reached 97.5%.

Apical and posterior segments of the upper lobes were the main localisation of lung lesions in most patients (82.5%), superior segments of the lover lobes rarely being damaged (17.5%). The main lesion in NTMPD and combination of NTMPD with pTB was often localized in apical and posterior segments of the upper lobes (35% and 56.5%, respectively). However, localisation in segments atypical for tuberculosis in NTMPD was also not uncommon. The main lesion in S4 and S5 was observed in 30% of patients in Group 1 and 8.7% Group 2. S6 suffered damage in 20% and 13% of patients respectively, S3 in 5% and 13%, and the basal segments in 10% and 8.7%.

There could be more than one radiographic sign for one patient (Table 7). In Group 1 nodule lesions were found in 10 (50%) patients, thin-walled cavities in 8 (40%), thick-walled cavities in 1 (5%), round shadows more than 1 cm in diameter with destruction in 5 (25%), without destruction in 2 (10%), multifocal bronchiectasis in combination with other manifestations in 4 (20%), lung infiltrates in 4 (20%), ground-glass areas in 1 (5%), pericissuritis in 1 (5%), volume reduction of the lung lobe or the lung in 4 (20%), thickening of the bronchial wall in 4 (20%), enlarged intrathoracic lung lymph nodes in 5 (25%) and pneumofibrosis in 2 (10%). Patients with XDR pTB had thin-walled cavities, nodule distribution along with volume reduction of the lung lobe or the lung and pneumofibosis in all cases. In addition, diffuse shadowing (infiltrations) indicated itself more commonly in Group 3 than in Groups 1 and 2. This was the primary difference between Group 3 and Groups 1 and 2. 

In total 72 patients had concomitant illnesses or conditions. A total of 180 cases of concomitant pathology were noted. Chronic bronchitis and chronic obstructive pulmonary disease were the most common conditions (41 patients). In Group 1, 2 patients had diseases of the gastrointestinal tract, in Group 2—4 and in Group 3—12. There was liver pathology in 4 patients of Group 1, in 7 of Group 2 and in 12 of Group 3. Heart diseases were identified in 6 patients in Group 1, 5 patients in Group 2 and 14 patients in Group 3.

Many patients had complications of underlying pulmonary process (Table 8). Most common were respiratory failure (RF) in 63 patients, specific involvement of the trachea, larynx, or bronchi in 14, and hemoptysis in 10 patients. The frequency of complications was compared, the frequency of hemoptysis and respiratory failure having statistically significant differences depending on the diagnosis (*p* = 0.027, *p* = 0.005, respectively). Significant differences between groups of patients were not revealed when analysing other complications of pulmonary process (*p* = 0.266, *p* = 0.145, *p* = 0.272, *p* = 0.203, *p* = 0.069, *p* = 0.245, respectively).

The frequency analysis of complaints (Table 9) for weakness, shortness of breath or hemoptysis depended on the final diagnosis and showed significant differences (*p* < 0.001, *p* < 0.001, *p* = 0.027, respectively). Shortness of breath and weakness were more common in patients with XDR pTB and hemoptysis than in patients with NTMPD. When assessing the incidence of cough, low-grade fever, pain in the chest, acute onset and loss of appetite, it was not possible to establish statistically significant differences between patients with different diagnoses (*p* = 0.080, *p* = 0.963, *p* = 0.668, *p* = 0.279, *p* = 0.324, respectively).

Overall, 156 operations were performed for 83 patients: 28 operations on 20 patients in Group 1, 41 on 23 patients in Group 2 and 87 on 40 patients in Group 3 (Table 10).

In Group 3 (XDR pTB) there were 4 (4.6%) wedge resections, 11 (12.6%) segmentomies, 10 (11.5%) lobectomies, 7 (8.0%) lobectomies with segmentectomy, 1 (1.1%) bilobectomy, 8 (9.2%) pneumonectomies, 5 (5.7%) transsternal occlusion of the main bronchus, 4 (4.6%) thoracocentesis, 31 (35.6%) thoracoplastics, 2 (2.3%) rethorcoplastics, 1 (1.1%) endobronchial valve and 3 (3.4%) others.

Out of 87 operations, 41 lung resections were performed, of which 26 (63.4%) were video-assisted thoracoscopic surgery (VATS). Out of 33 thoracoplasties and rethoracoplasties, 28 (84.8%) were also performed through VATS access using the original technique [1]. Generally, VATS was performed during 54 (62.1%) operations in Group 3.

## 4. Discussion

Patients of all three groups had a high incidence of lung cavitary lesions (70% in Group 1, 100% in Group 2 and 100% in Group 3). The difference between patients with NTMPD and pTB cannot be evaluated since the presence of cavitary lesions was the defining criteria for Group 3; this does not contradict many authors who note that cavitary lesion is common among patients with NTMPD [34,35,36]. The presence of complications of the pulmonary process (50.0%; 73.9%; 60.0%, respectively), respiratory failure (50.0%; 91.3%; 80.0%, respectively), concomitant diseases (80.0%; 87.0%; 90.0%, respectively), preservation of bacterial excretion before surgery (50%; 73.9%; 97.5%, respectively) and the presence of multiple and extensive drug resistance among patients who were able to test for DR (100%; 93.3%; 100%). NTMPD clinical signs were less severe than XDR pTB as seen in Table 8. Clinical manifestations in patients with a combination of NTMPD and pTB were less severe than in Group 3, but more so than in Group 1. Such findings were expected. They further demonstrated that, clinically, the signs and symptoms of NTMPD are varied and nonspecific. This does not contradict most existing publications [37,38,39,40]. 

However, symptoms such as hemoptysis occurred much more frequently in Group 1 and Group 2 in comparison with Group 3. It is well known that hemoptysis is common among patients with pTB [41,42,43,44,45]. On the other hand, there are not too many trials that investigate hemoptysis among patients with NTMPD [46,47], and we do not find any among patients with a combination of NTMPD and pTB. It is important to note that there is at a minimum one trial where the incidence of hemoptysis was not significantly different between patients with pTB and NTMPD [48]. The lower severity of the clinical picture for Group 2 compared with Group 3 was apparently associated with a longer duration of the disease and a more severe clinical form of tuberculosis (fibrous-cavernous in all patients of Group 3) (Table 3).

Group 1 has the greatest variance in radiographic features. Patients with XDR pTB had no such variation. In patients with a combination of NTMPD and pTB, the radiographic picture included the same range of change as in patients of Groups 1 and 3. However, despite most authors noting a similar radiographic picture of tuberculosis and NTMPD [34,49,50], in 14 (32.6%) patients of Groups 1 and 2, multifocal bronchiectasis was detected, while in the group of patients with XDR pTB it occured 13 times less frequently and was found only in 1 (2.5%) patient. The presence of bronchiectasis allows for possible mycobacterial infection, and the presence of nodule distribution with severe pneumofibrosis is more common in tuberculosis [50,51,52,53,54,55].

For most patients in Groups 1 and 2, pathological processes in the lungs were unilateral (65% and 73%, respectively), while bilateral processes prevailed (62%) in Group 3. A limited (up to three segments) process was observed in half of the cases for patients of Group 1, whereas in Group 2, the majority (82.6%) of patients had a widespread pulmonary process. In Group 3, the frequency of widespread lung damage constituted 97.5% of patients.

The primary lung lesions of pTB were commonly localised in the apex and posterior segments of the superior lung lobe (82.5%). Localization in S6 is uncommon (17.5%). The primary lung lesions of NTMPD and a combination of NTMPD and pTB were also commonly localised in the apex and posterior segments of the superior lung lobe (35% and 56.5%, respectively). However, localisation in segments atypical for tuberculosis was not rare (S5 in 30% of patients of Group 1 and 8.7% of Group 2; S3 in 5% and 13%, respectively; basal segments—10% and 8.7% respectively).

Our results show that there is no pathognomonic radiographic appearance in Groups 1 and 2. The disease was usually unilateral. A similar distribution of infection was reported previously [56,57].

In addition, we would like to note that some Group 2 patients had long courses of chemotherapy prior to surgery, of duration longer than 6 months. One possible reason for this is the varied course of disease. Other reasons include drug resistance, bad compliance and that the TB treatment could be not effective in suppressing both TB and NTMPD simultaneously, along with other undetermined causes.

Weaknesses in our study are its retrospective nature and a relatively small number of observations. This does not allow us to draw definitive conclusions. Despite this, the rarity of NTMPD and its diagnostic difficulties obliges us to share our experiences with the wider medical community.

## 5. Conclusions

From the data, patients with NTMPD have a higher frequency of pulmonary haemorrhages than those without NTMPD. This is evident from the higher incidence of pulmonary haemorrhages in Group 1 (25%) and Group 2 (17.4%) patients compared with Group 3 (2.5%). In Group 3 there was the greatest proportion of pathological processes per patient (the number of lung cavitary lesions, the frequency of bilateral lesions and the number of affected lung segments). Group 1 had substantially lower numbers. The combination of NTMPD and pTB (Group 2) occupied an intermediate position in terms of prevalence and severity of such processes, being significantly higher than those of Group 1.

Thus, in the clinical picture of the combination NTMPD and pTB, there are no pathognomonic clinical and radiographic symptoms, which is noted by most authors [2,3,4,5]. However, the presence of an indolent course, hemoptysis and bronchiectasis in the presence of MBT in the sputum makes it possible to suspect a combination of NTMPD and pTB, which requires in-depth bacteriological examination and, additionally, more careful re-examination for radiographic and clinical signs. In 12 patients (52.2%) of the second group, we managed to make the correct diagnosis only after the operation, taking into account the data of the morphological and bacteriological study of the operative material.

## Figures and Tables

**Table 1:** Distribution of patients by gender.

Gender	Group 1	Group 2	Group 3	*p*
female	10 (50.0%)	14 (60.9%)	13 (32.5%)	0.079
male	10 (50.0%)	9 (39.1%)	27 (67.5%)	

**Table 2:** Analysis of age at first admission in compared groups.

	Age at Admission (Years)			***p***
	M ± SD	95% CI	n	
Group 1	34 ± 14	28–40	20	0.361
Group 2	36 ± 11	31–40	23	
Group 3	31 ± 11	28–35	40	

**Table 3:** Average disease duration before surgery.

	Average Disease Duration before Surgery (Months)			*p*
	Me	Q₁–Q₃	n	
Group 1	6	3–11	20	<0.001 * p_group3-group1_ < 0.001 *p*_group3-group2_ < 0.001
Group 2	12	8–18	23	
Group 3	36	13–78	40	

*—differences in indicators are statistically significant (*p* < 0.05)

**Table 4:** Max cavitary lesion size.

		Group 1	Group 2	Group 3	*p*
Max cavitary lesions size	no cavities	6 (30.0%)	0	0	<0.001 * p_Group1-Group3_ = 0.003
	<2 cm	0	1 (4.3%)	1 (2.5%)	
	2–4 cm	13 (65.0%)	17 (73.9%)	25 (62.5%)	
	4.1–6 cm	1 (5.0%)	2 (8.7%)	3 (7.5%)	
	>6 cm	0	3 (13.0%)	11 (27.5%)	

*—differences in indicators are statistically significant (*p* < 0.05)

**Table 5:** Frequency of unilateral and bilateral pulmonary destruction.

Lung Destructions	Patients with Unilateral Cavities			Patients with Bilateral Cavities			All Patients with Cavities
	Group 1	Group 2	Group 3	Group 1	Group 2	Group 3	
single cavitary lesions	8 (57.1%)	14 (60.8%)	10 (25%)	0	0	0	32 (38.6%)
multiply cavitary lesions	4 (28.6%)	8 (34.7%)	15 (37.5%)	2 (14.3%)	1 (4.4%)	15 (37.5%)	45 (54.2%)
all patients with cavitary lesions	12 (85.7%)	22 (95.6%)	25 (62.5%)	2 (14.3%)	1 (4.4%)	15 (37.5%)	77 (92.8%)

*—differences in indicators are statistically significant (*p* < 0.05)

**Table 6:** Main radiographic signs at admission.

		Group 1	Group 2	Group 3	*p*
The main radiographic sign upon admission	lung focal lesions less than 1 cm in diameter without destructions	2 (10.0%)	0	0	<0.001 * p_Group1-Group3_ < 0.001
	lung focal lesions more than 1 cm in diameter without destruction	2 (10.0%)	0	0	
	lung focal lesions more than 1 cm with destructions	5 (25.0%)	3 (13.0%)	0	
	thick-walled cavitary lesions	1 (5.0%)	5 (21.7%)	0	
	thin-walled cavitary lesions	8 (40.0%)	15 (65.2%)	40 (100.0%)	
	bronchiectasis	2 (10.0%)	0	0	

*—differences in indicators are statistically significant (*p* < 0.05)

**Table 7:** All radiographic signs at admission.

Size and Localization of Affected Lung Parts	Number of Cases			*p*
	Group 1	Group 2	Group 3	
unilateral pathological changes	13 (65.0%)	17 (73.9%)	15 (37.5%)	0.011 * p_group2-group3_ = 0.016
bilateral pathological changes	7 (35.0%)	6 (26.1%)	25 (62.5%)	
localized pathological changes (3 or less lung segments)	10 (50.0%)	4 (17.4%)	1 (2.5%)	0.005 * p_group1-group3_ = 0.002
wide pathological changes (more than 3 lung segments)	10 (50.0%)	19 (82.6%)	39 (97.5%)	
damage to s1, s2	7 (35.0%)	13 (56.5%)	33 (82.5%)	
damage to s6	4 (20.0%)	3 (13.0%)	7 (17.5%)	
damage to 3s	1 (5.0%)	3 (13.0%)	0	
damage to s4, s5	6 (30.0%)	2 (8.7%)	0	
damage to s7–s10	2 (10.0%)	2 (8.7%)	0	
ground-glass opacity	1 (5.0%)	1 (4.3%)	0	
diffuse shadowing (infiltrations)	4 (20.0%)	3 (13.0%)	11 (27.5%)	
round shadow less than 1 cm in diameter (nodules)	10 (50.0%)	15 (65.2%)	40 (100.0%)	0.001 * p_group1-group2_ = 0.049 *p*_group1-group3_ = 0.010
round shadow in the lung more than 1 cm without destruction (tuberculoma)	2 (10.0%)	0	0	0.040 *
shadow in the lung more than 1 cm with destruction (tuberculoma)	5 (25.0%)	3 (13.0%)	0	0.007 * p_group1-group3_ = 0.003 *p*_group2-group3_ = 0.039
thin-walled cavitary lesions	1 (5.0%)	5 (21.7%)	0	0.005 * p_group2-group3_ = 0.006
thick-walled cavitary lesions (fibrotic cavities)	8 (40.0%)	15 (65.2%)	40 (100.0%)	<0.001 * p_group1-group3_ < 0.001 *p*_group2-group3_ < 0.001
periscissuritis (regional infiltration) **	1 (5.0%)	4 (17.4%)	8 (20.0%)	
volume reduction of the lung lobe or the lung	4 (20.0%)	13 (56.5%)	40 (100.0%)	
multifocal bronchiectasis	4 (20.0%)	10 (43.5%)	1 (2.5%)	
thickening of the bronchial wall	4 (20.0%)	8 (34.8%)	9 (22.5%)	
enlarged intrathoracic lung lymph nodes	5 (25.0%)	8 (34.8%)	16 (40.0%)	
cirrhotic changes (pneumofibrosis)	2 (10.0%)	9 (39.1%)	40 (100.0%)	
thickening of the pleural sheets, pleural exudate	0	4 (17.4%)	4 (10.0%)	0.069

*—differences in indicators are statistically significant (*p* < 0.05); ** Periscissuritis of the triangle form is cloudy infiltration, located at the interlobe fissure. The tip of a triangle is inverted to lung root, base to peripheral. The top border is undefined and cloudy passing without sharp outlines in unchanged lung tissue. The bottom border corresponds to inter-lobe pleura and is consequently precise. On the tomogram, the background has inflammatory infiltrations which differentiate the shadows of more or less dense foci, cavities of disintegration, and rod and scar formations of condensed parenchyma.

**Table 8:** Characteristics and incidence of pulmonary process concomitant illnesses.

Complications Type	Number of Complications		
	Group 1, n = 20	Group 2, n = 23	Group 3, n = 40
respiratory failure	10 (50.0%)	21 (91.3%)	32 (80.0%)
hemoptysis, pulmonary haemorrhage (0.027 * *p*_group1-group3_ = 0.019)	5 (25.0%)	4 (17.4%)	1 (2.5%)
spontaneous pneumothorax	1 (5.0%)	0	0
empyema	0 (0.0)	3 (13.0%)	4 (10.0%)
bronchostenosis	0	3 (13.0%)	5 (12.5%)
specific damage to the larynx, trachea, or bronchi (*p* = 0.069)	0	5 (21.7%)	9 (22.5%)
aspergillosis	1 (5.0%)	0	0
total patients with complications	10 (50.0%)	21 (91.3%)	35 (87.5%)

*—differences in indicators are statistically significant (*p* < 0.05)

**Table 9:** Analysis of complaints depending on final diagnosis.

Complaint	Number of Complaints			
	Group 1	Group 2	Group 3	*p*
weakness	11 (55.0%)	13 (56.5%)	39 (97.5%)	<0.001 * p_group1-group3_ = 0.001 *p*_group2-group3_ < 0.001
cough	8 (40.0%)	13 (56.5%)	28 (70.0%)	0.080
dyspnea	4 (20.0%)	6 (26.1%)	26 (65.0%)	<0.001 * p_group1-group3_ = 0.003 *p*_group2-group3_ = 0.006
subfebrile condition	6 (30.0%)	7 (30.4%)	11 (27.5%)	0.963
hemoptysis, pulmonary haemorrhage	5 (25.0%)	4 (17.4%)	1 (2.5%)	0.027 * p_group1-group3_ = 0.019
chest pain	1 (5.0%)	3 (13.0%)	4 (10.0%)	0.668
acute onset	1 (5.0%)	1 (4.3%)	6 (15.0%)	0.279
loss of appetite	5 (25.0%)	9 (39.1%)	18 (45.0%)	0.324
total patients with complaints	14 (70.0%)	22 (95,7%)	40 (100%)	

*—differences in indicators are statistically significant (*p* < 0.05)

**Table 10:** Types of operations.

Surgery Type	Group 1		Group 2		Group 3		Total/of Which Minimally Invasive Access
	Total	VATS *	Total	VATS *	Total	VATS *	
wedge resection	14 (50.0%)	14	8 (19.5%)	8	4 (4.6%)	4	26/26
segmentectomy	5 (17.9%)	3	0	0	11 (12.6%)	11	16/14
lobectomy with segmentectomy	2 (7.1%)	0	5 (12.2%)	4	7 (8.0%)	5	14/9
lobectomy	0	0	6 (14.6%)	3	10 (11.5%)	6	16/9
bilobectomy	0	0	2 (4.9%)	2	1 (1.1%)	0	3/2
pneumonectomy	1 (3.6%)	0	3 (7.3%)	0	8 (9.2%)	0	12/0
transsternal occlusion of the main bronchus	0	0	0	0	5 (5.7%)	0	5/0
thoracocentesis	3 (10.7%)		2 (4.9%)		4 (4.6%)		9/9
pleurectomy with wedge resection	0	0	1 (2.4%)	1	0	0	1/1
thoracoplasty	3 (10.7%)	3	13 (31.7%)	11	31 (35.6%)	26	47/40
rethoracoplasty	0	0	0	0	2 (2.3%)	2	2/2
endobronchial valve	0		0		1 (1.1%)		1/1
others	0	0	1 (2.4%)		3 (3.4%)		4/4
total number of operations:	28 (100.0%)	20 (71.4%)	41 (100.0%)	29 (70.7%)	87 (100.0%)	54 (62.1%)	156 (100%)/ 117(75.0%)

* VATS–Video-assisted thoracoscopic surgery.

## Data Availability

The data presented in this study are available on request from the corresponding author.

## Supplementary Materials

The following supporting information can be downloaded at: https://www.mdpi.com/article/10.3390/pathogens12070887/s1, Table S1: Patients’ characteristics.
